# Detection of Mechanical Failures in Industrial Machines Using Overlapping Acoustic Anomalies: A Systematic Literature Review

**DOI:** 10.3390/s22103888

**Published:** 2022-05-20

**Authors:** Ahmad Qurthobi, Rytis Maskeliūnas, Robertas Damaševičius

**Affiliations:** Faculty of Informatics, Kaunas University of Technology, 44249 Kaunas, Lithuania; ahmad.qurthobi@ktu.edu (A.Q.); rytis.maskeliunas@ktu.lt (R.M.)

**Keywords:** acoustic recognition, mechanical failure, industrial machines, systematic review

## Abstract

One of the most important strategies for preventative factory maintenance is anomaly detection without the need for dedicated sensors for each industrial unit. The implementation of sound-data-based anomaly detection is an unduly complicated process since factory-collected sound data are frequently corrupted and affected by ordinary production noises. The use of acoustic methods to detect the irregularities in systems has a long history. Unfortunately, limited reference to the implementation of the acoustic approach could be found in the failure detection of industrial machines. This paper presents a systematic review of acoustic approaches in mechanical failure detection in terms of recent implementations and structural extensions. The 52 articles are selected from IEEEXplore, Science Direct and Springer Link databases following the PRISMA methodology for performing systematic literature reviews. The study identifies the research gaps while considering the potential in responding to the challenges of the mechanical failure detection of industrial machines. The results of this study reveal that the use of acoustic emission is still dominant in the research community. In addition, based on the 52 selected articles, research that discusses failure detection in noisy conditions is still very limited and shows that it will still be a challenge in the future.

## 1. Introduction

During collection, compression, and transmission, all collected signals and acquired images are unavoidably polluted by noise, resulting in distortion and loss of information. The quality of any signal processing activities is harmed by the presence of noise. As a result, signal denoising is critical in today’s signal processing systems, such as related to image processing [1], speech recognition [2], or biomedical signal processing for medical diagnostics [3]. In telecommunication, noise reduces the bandwidth of communication channels and leads to signal jitter and information loss [4]. In urban environments, noise affects negatively the health of citizens and leads to noise pollution [5]. Noise is also harmful in many industrial applications and construction engineering [6]. Industrial noise is acoustic noise that occurs at workplaces and enterprises as a result of the production process, during the operation of machines, equipment, and tools [7]. The result of industrial noise leads to a reduced lifetime of industrial machinery and/or industrial accidents. Structural vibration, which is conceptually similar to noise, can cause many noise-related problems: it can cause structural fatigue failure [8], cause discomfort to people using the product or bystanders [9], disrupt sensitive equipment, and so on [10]. The crucial initial stage in the actual engineering application of unit condition monitoring and fault diagnosis is to analyze vibration data in order to extract the most representative problem characteristics and increase the accuracy of diagnosis and analysis. As a result, efficient noise analysis of the gathered vibration signals is critical for properly judging the unit’s defective function.

In the large-scale industries, where plentiful industrial machines are involved, not every occurrence of mechanical failure on every single machine could be directly detected by commonly used sensors [11,12,13]. One of the causes of this disability is triggered by the high level of noise in the environment in which the machines are operated. In a very noisy condition, whether caused by light or sound pollution, the commonly used sensors, e.g., ultrasonic and infra-red sensors, will experience a large amount of distortion and encounter difficulties in disturbance or failure detection.

Several studies have been conducted to detect failures in industrial machines [14]. For example, deep-learning-based anomaly detection, a new detection method in another area of signal processing [15,16], can be used to detect such failures. In addition, one method that is also commonly used to detect mechanical damage to machines is the acoustic method. This method is used because it has a higher level of security compared to other methods, because measurements do not have to be performed via direct contact with the monitored equipment [17]. The use of acoustic methods to detect the irregularities in systems has a long history [18,19]. In general, the abnormal conditions that occur at the measured device or location can be detected by changes in the characteristics of the acoustic signal generated, such as frequencies and amplitude [20,21]. The advantage of using the acoustic method compared to other methods is that the features of the acoustic signal can be extracted and used for deeper failure detection [22]. Moreover, the use of acoustic methods is also applied to detect changes in the behavior of living creatures [23]. Considering the growing interest of the research community in the detection of failures by acoustic methods in general, and, in particular, failures in high-noise environments, a large number of relevant methods and equipment have emerged over the last few years. Several secondary studies have provided scope for this solution, but systematic studies in this research domain are still very limited.

The main contribution of this research is the systematic literature review (SLR) that was used to analyze and synthesize the relevant studies on failure detection by acoustic methods, and the technology that has been used and will be used for failure detection by acoustic methods. This research also aims to investigate the primary techniques and algorithms for acoustic-based failure detection, as well as to identify several methods that demonstrate the potential for using these techniques. This study also discusses several taxonomies. This study used an evidence-based systematic review methodology to cover the most recent literature and to follow a systematic and impartial selection and evaluation process as a form of transparency and to ensure the inclusion of all related studies.

The main purposes of this study are:

1. Classifying acoustic mechanical failure analysis approaches and techniques;
2. Analyzing the existing work conducted in this area of research;
3. Recognizing the main issues that need to be handled;
4. Identifying the potential areas of research in the future.

## 2. Related Work

This section presents a brief discussion of the relevant literature review and research on the detection of mechanical failures using acoustic methods. Table 1 shows a comparison of reviews and surveys on it. Delvecchio et al. [24] wrote a critical review of the use of the vibro-acoustic method to monitor internal combustion engines (ICM). Leaman et al. [25] wrote a review on using acoustic emission technology to detect failures in planetary gearboxes (PG). Lukonge and Cao [26] wrote a review on the utilization of acoustic emissions technology to detect offshore and onshore pipeline leaks. Raghav and Sharma [27] presented a review on condition monitoring techniques and fault and failure diagnosis on a gearbox based on the acoustic emission (AE) method.

Reviews were conducted and reported using the guidelines for systematic literature reviews and the systematic mapping study process and the Preferred Report Items for Systematic Reviews and Meta-Analysis statements (PRISMA). This systematic review is based on a well-designed research process that ensures the comprehensive and impartial selection of all peer-reviewed publications related to published research material. This protocol is used to collect relevant papers from credible scientific sources, which are then classified and mapped into several categories to reveal the true state of the ongoing research in the application of failure detection technology. This research map will be very useful for practitioners and researchers in determining state-of-the-art domains and topics for future research.

Consequently, it is important to note that the aim of this review is not only to identify use cases or applications of acoustic methods to detect failures, but also to understand the limitations and challenges of using such methods. In addition, we examine the latest trends in terms of technical approaches, methodologies, and concepts used in the implementation of these methods.

## 3. Research Methodology

The goal of using an SLR is to distinguish, evaluate, and examine previous and related works that are relevant to the purpose of this paper. Reviewing studies with a logical and impartial research approach can result in SLR writing. The research strategy must be capable of ensuring the completion of the evaluation procedure as soon as possible, according to Kitchenham. Nonetheless, the primary goal of running an SLR is to fill in the gaps that exist in each area. Furthermore, the unique nature of this systematic review necessitates similar research to serve as a guide.

### 3.1. Research Design

In this subsection, the current research requirements are described by identifying the results of the preliminary research based on the research question and keywords related to the research question.

#### 3.1.1. Literature Review Questions

It has taken a long time to develop methods for identifying and illustrating failure detection methods using acoustic methods. Various processes, methodologies, and techniques have been developed over the years to describe the elements involved in acoustical failure detection. As a result, the following questions will be addressed in this study:

1. What types of failures in industrial machines can be detected by acoustic methods?
2. What are the existing solutions and possible technologies for the detection of mechanical failures by acoustic methods?
3. What are the challenges faced by acoustical failure detection?
4. What are the future research trends and directions in mechanical failure detection using acoustic methods?

#### 3.1.2. Research Process

Rather than resources drawn from scientific articles, this literature review process focuses on finding accredited main study articles. Furthermore, scientific conference proceedings are regarded as research sources. To continue the process of extracting SLR review articles, the following resources were used.

#### 3.1.3. Search Terms

Several online database sources were involved to search for and collect papers related to this study. These sources were selected based on the establishment they have achieved to date. The sources of the papers used as references in this study can be seen in Table 2. This database can provide the highest impact and full text of the most important journals and conferences relevant to acoustical failure detection.

After performing the first search step by entering keywords in this database, an additional scanning step was performed to ensure the accuracy of the research process and that the selection of studies relevant to the current research question and work met the criteria. In this study, search engines were also involved to assist the search process for related research.

1. “Acoustic Mechanical Failure Detection Industrial Machine” OR “Acoustic Mechanical Fault Detection Industrial Machine”
2. “Acoustic Mechanical Failure”
3. “Acoustic Detection”
4. “Acoustic”
5. “Mechanic Failure”
6. Detection
7. Failure
8. Machine

The search terms were then aggregated into a search query using conjunction (AND) and disjunction (OR) operators.

(((((“Industrial Machine”) AND “mechanical”) AND “Failure” OR “Fault”) AND “Acoustic”) AND “Detection”)

### 3.2. Review Conduction

This section describes the approaches involved in carrying out the systematic literature review process. The SLR search process depends on the rules and frameworks involved in producing this review article.

#### 3.2.1. Selection of Relevant Papers

**Figure 1:**
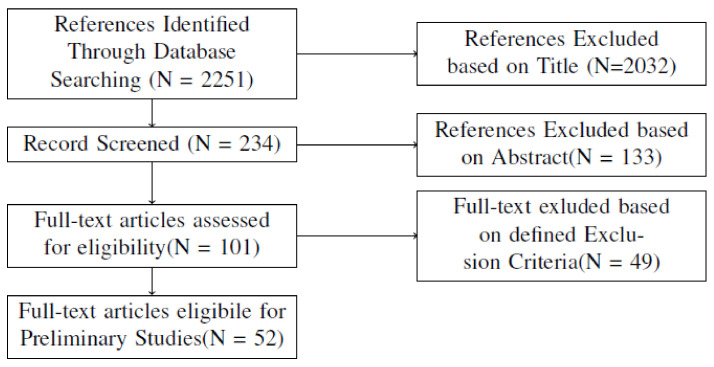
Procedure of research selection for the present schematic review.

Following the acquisition of preliminary research studies related to the research objectives, the discovered papers should be evaluated for relevance. As a result, a second assessment was carried out in order to determine the relevance of the chosen initial study through an evaluation. In addition, after the initial screening, a systematic review of the selected studies was performed at random to ensure the consistency of the inclusion and exclusion criteria. Figure 1 depicts the study selection procedure for the current systematic review.

The following steps were taken to identify relevant research studies:

1. Find the database and identify previous works related to the study using the defined terms.
2. Ignore papers that are not related to the given search criteria.
3. Exclude papers that have no clear relationship between title or abstract.
4. Evaluate the papers by reading the full context.
5. Evaluate the bibliography
6. Perform the initial study.

#### 3.2.2. Inclusion and Exclusion Criteria

**Figure 2:**
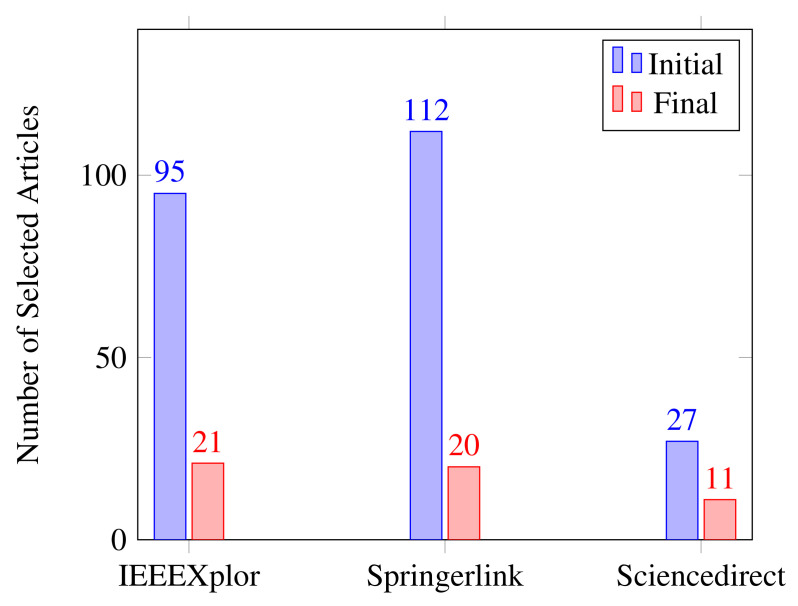
Proportion of selected studies.

Exclusion criteria included research articles that were not related to an acoustic approach to detecting mechanical failure and were therefore outside the scope of this research paper. This research focused on SLR research articles that were relevant to this topic. Furthermore, similar studies on the same topic were not included in the study. As a result, Table 3 shows the inclusion and exclusion criteria used in writing the SLR. Figure 2 depicts the proportions of initial and final article selections from each online source listed in Table 3.

#### 3.2.3. Data Extraction

Relevant information was extracted from the articles during the data extraction process and placed into a database. This database consisted of the items listed in Table 4.

### 3.3. Demographic Data and Overview

The results of the systematic review are reported in this section. As shown in Figure 1, 2251 documents were extracted from the scientific database using a search methodology. In total, 2032 papers were eliminated after the initial screening, which was based on the article title and keywords, leaving 233 for additional screening. The publication did not commit to discussing the use of acoustic methods to detect failures; however, the content of the abstract, considered to be related to the method, led to the search protocol used to be included in the list of related publications. After reading the abstracts of the selected articles, as well as the introduction and conclusions in some cases, in the following screening stage, we screened the papers further using the criteria stated in Table 3. A total of 101 papers were selected as a consequence of this process. Another 49 articles were eliminated after reading all selected papers because they did not focus on detecting failures in industrial machines. After the screening procedure, 52 publications were selected for inclusion in the study. Table 5 contains a complete list of the selected publications, as well as some of the data elements retrieved.

**Figure 3:**
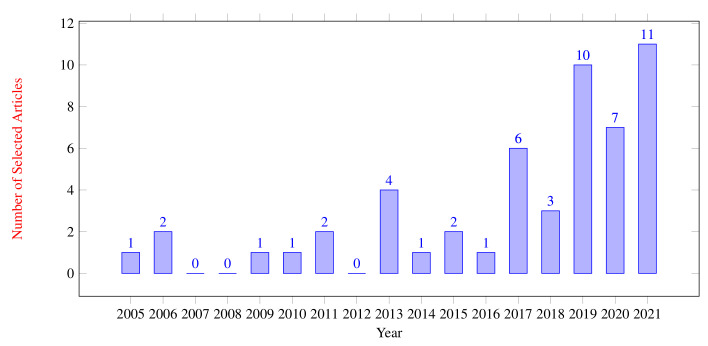
Year of publication.

The time span of the articles used is from 2006 to 2021. Figure 3 shows the distribution of the included articles, with most of the articles (11 articles) published in 2021. Of the 52 included papers, 37 were published in the last five years (2017–2021). This implies that research in the field of acoustical methods for detecting failures is still very new and interest in this area is growing rapidly as the number of publications continues to increase.

**Figure 4:**
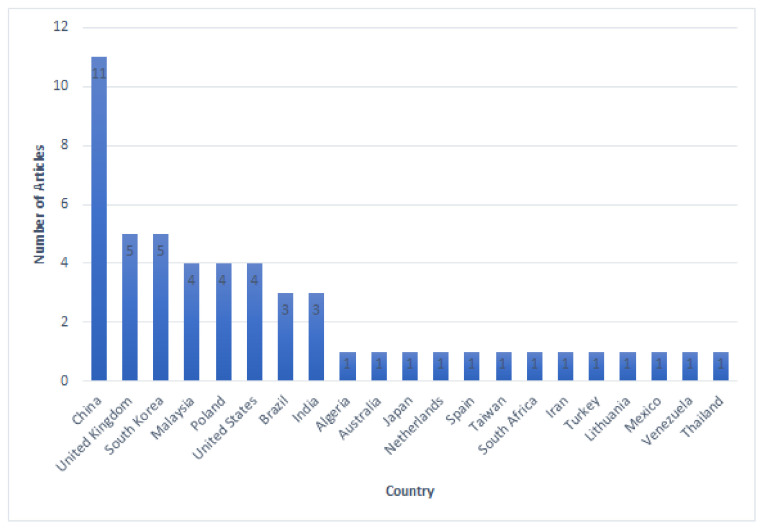
Article distribution by country of origin.

The location (country) of the institutions associated with the authors of the selected publications was also used to obtain an overview of the geographical distribution of members of the research community interested in research on acoustic methods for failure detection. The institution of origin of the author of the correspondence, or the first author if the author of the correspondence is unknown, is determined as the country of origin of the selected article. The geographical distribution of the article authors is shown in Figure 4. Based on the 52 articles reviewed, China was the largest contributor, with 11 articles, followed by South Korea and the United Kingdom with five articles. Malaysia, Poland, and the United States followed in the next position, with each contributing four articles. Meanwhile, Brazil and India contributed three articles, and 13 different countries contributed one article each. 

**Figure 5:**
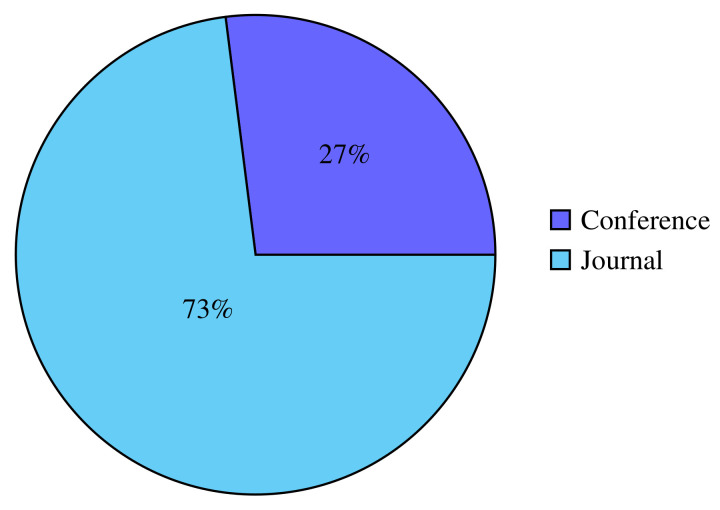
Categories of publication.

The type of publication determines whether the paper will be published in journals and conferences. The publishing categories of the publications collected are depicted in Figure 5. In this study, 73% or 38 of the selected articles came from publications in the form of scientific journals. The rest, 27% or 14 of the selected articles, came from scientific conferences. The list of journals and conferences that become publication media can be seen in Table 6.

## 4. Results and Discussion

### 4.1. Results Obtained from Answering the Research Questions

In this section, the results obtained are the answers to the research questions given in Section 3.1.1. These questions were asked to determine the extent of research developments in the field of using acoustic methods to detect failures in industrial machines. The answers to these questions are compiled based on the description of the results of the selected scientific articles.

#### 4.1.1. What Types of Failures in Industrial Machines Can Be Detected by Acoustic Methods?

Table 7 shows the types of damage to industrial machines that can be detected by the acoustic method. The table shows that, based on the collected references, mechanical failures that can be found by the acoustic method include defects, wear, fractures, leaks, and others. Grinding burn is caused by excessive heat generated during the grinding process. Gao et al. [36] designed a grinding burn monitoring system using acoustic emission signals and wavelet coherence analysis. Breakage is another example of a failure that can be detected by acoustic methods, as shown in research performed by Sun et al. [71] involving mechanical breakage analysis on milling machines. Other failure types that can also be found by the acoustic method, based on the selected articles, are corrosion [28], cracks [57,72], leakage [30,52], wear [46,55], rubbing [62], pitting [53], etc. Table 7 also shows that most of the detected failures were in bearings (17 articles) and gears (12 articles). This shows that the detection of failures with the acoustic method is very suitable for use on components that have a high level of movement.

In general, mechanical failure is a failure type that causes disruption or cessation of the work of a device. This failure can be caused by cracks [75], deformation, wear [46,47], leakage [30], bending, etc. Mechanical failure can be recognized by the increase in temperature or the appearance of an unusual sound when the engine is operating [80].

#### 4.1.2. What Are the Existing Solutions and Possible Technologies for the Detection of Mechanical Failures by Acoustic Methods?

In the field of acoustics, mechanical failure can be recognized by the appearance of an unusual signal when the engine is operating. This damage signal generally has a frequency and amplitude that are not the same as the frequency and amplitude under normal conditions. According to Tagawa et al. [22], acoustic data are easier to collect at the factory due to the relatively low cost of installing microphones in existing facilities.

Broadly speaking, the use of acoustic methods to detect mechanical failures in machines can be divided into two categories, namely the utilization of acoustic emission and the others (see Table 8). The acoustic emission method is the most commonly used acoustic analysis method in detecting mechanical failure. On the other hand, the other methods harvest the acoustic signal by utilizing a common sound sensor such as a microphone.

- Acoustic Emission-Based
  Acoustic emission (AE) is the term given to describe a physical phenomenon that occurs when a small amount of elastic energy is released into a structure through a mechanical process [20]. In simple terms, the acoustic emission signal is a combination of the deterministic signal and the failure signal. A deterministic signal is a signal that appears when the engine is running normally. Meanwhile, the failure signal is a signal that appears when there is an abnormality or disturbance when the engine is operating. Assuming that the deterministic signal and the failure signal are unrelated, Liu et al. [50] write the acoustic emission signal as Equation (1), where y(n), d(n), and ξ(n) are, respectively, acoustic emission signals, deterministic signals, and fault signals.
  (1) $$\begin{array}{c} y(n)=d(n)+\xi (n)\;\mathrm{for}\;n=1,2,\cdots ,M+N \end{array}$$
- Microphone-Based
  Apart from the acoustic emission approach, there are various other ways to retrieve the acoustic signal from the component to be inspected. In general, acoustic signal retrieval involves using a microphone to pick up the signal. The microphone used can stand alone [22,47], with additional equipment involvement (such as a stethoscope) [78], or a microphone may be used that is installed on certain devices (such as cellphones) [60,67]. The use of a microphone is intended to take sound samples from the device under test when the equipment is working in accordance with its function. The frequency of the sound picked up by the microphone can be in the range of 10 Hz–10 kHz (the range of sound that can be heard by humans) [59], as well as the signals picked up by the microphone on a mobile phone sampling frequency of 44.1 kHz [47,67]. The advantage of using a microphone over other methods is the ease of installation and data collection [22]. However, careless placement of the microphone will affect the measurement results.
- Ultrasonic-Based
  Another method used to detect faults is to utilize ultrasonic signals. Jo et al. [45] conducted research on failure detection on turbine blades by the ultrasonic method at a frequency of 300 kHz. They found that partially lost and distorted blades can be detected by acoustic diagnosis during the turbine’s operation.

Table 8 also shows that analysis using machine learning is the most preferred choice in determining failures with acoustic methods, both in acoustic emission-based studies and with microphones. Artificial neural networks, k-Nearest Neighbors, and SVM are the most common types of machine learning used in these studies. The use of these methods results in a detection system with an accuracy rate ranging from 80% to 100% [30,34,47,64,72].

Table 9 shows a list of examples of intelligent and classic methods used to determine failure in machines. On the other hand, Table 10 presents selected studies employing machine learning to perform machine failure detection. Both tables show that artificial intelligence in acoustic systems is still an attractive option for researchers.

#### 4.1.3. What Are the Challenges Faced by Acoustical Failure Detection?

At first, determining the failure that occurs in industrial machines without stopping the process is difficult. However, with the development of sensor technology, measurement, and computing, these problems have been overcome.

Industrial machine failures can occur in any machine or machine part. Failures can occur in bearings, gears, actuators, distributors, and others. With acoustic technology, failures can be measured even without the need for industrial process shutdowns, if needed, affordably and efficiently. This technology is very useful, especially for detecting early failures so that problems that occur can be handled immediately. However, behind these advantages, there are several challenges that must be faced in the application of the acoustic method. Table 11 aims to describe some of the problems encountered in the application of the acoustic method to detect failures.

#### 4.1.4. What Are the Future Research Trends and Directions in Mechanical Failure Detection Using the Acoustic Method?

Based on the review and investigation of more than 100 articles, various research directions and possible research topics for consideration for further research have been generated.

First, the use of acoustic emission methods still dominates research in the field of acoustic-based failure detection. This shows that there are still many opportunities to find new methods for such detection. Furthermore, as hardware and software technology advances, the opportunity to discover new methods will be even greater.

Second, there is still little research on the detection of mechanical failure with acoustic methods in a certain level of environment. Most of the research conducted is research on a laboratory scale. This shows the opportunity to conduct research for certain cases that are still wide open. Moreover, in actual conditions, the noise level will affect the results of data acquisition by the acoustic method.

Third, technological advances have led to increasingly sophisticated hardware specifications on devices such as mobile phones. Research initiatives in this regard are still very limited and can be taken as a future direction for portable failure detection devices. Extraction results from voice signal recordings on cell phones have been widely used for forensic purposes. Therefore, the use of mobile phones to replace existing sensors will remain an interesting discussion in the future.

Fourth, the use of artificial intelligence as a tool to analyze mechanical failures with acoustic methods is increasingly being selected. However, this does not rule out the possibility of implementing and developing other artificial intelligence algorithms for the failure detection case. Moreover, based on the reviewed papers, changes in location and the type of failure in equipment often require different analysis patterns.

Fifth, research on mechanical failure in industrial machines basically cannot be separated from research on work safety. Whenever there is an acoustically detected failure of an industrial machine, the control system must be able to set off an alarm with a certain level of vigilance. Therefore, it is necessary to conduct research that combines failure detection, severity, and decision making regarding the attitude that must be taken when the failure occurs in real time and centrally.

Lastly, the studies that have been done previously are generally only for detecting failures on individual machines. Research towards the detection of cumulative machine failures needs to be done. This is caused by the placement of machines in bulk in a room. Therefore, the design of a failure detection system for multi-device cases will be an interesting topic in the future.

### 4.2. Threats to Validity

Bias in the publication or selection process, errors in data extraction, and underestimation can undermine any systematic mapping research process.

The tendency of researchers to publish more positive results than negative results is known as publication bias. Positive results are more likely to be approved for publication and referred to by others. From a reviewer’s point of view, it is difficult to overcome publication bias. However, an attempt to overcome this has been made by scanning various respected scientific databases to find as many relevant papers as possible. As a result, several articles with positive results were eliminated and several studies with unsatisfactory results were published. However, by limiting the search of articles according to this method, there is a risk of neglecting important articles, such as reports from industry authorities. However, limiting the use of publications from selected databases is expected to increase the chances of finding high-quality scientific publications.

Selection bias, on the other hand, is more influenced by reviewers as it involves a tendency to leave certain relevant articles out of the analysis due to faulty search techniques. In this study, an attempt to create a search strategy was carried out and the results showed that it was able to find every relevant document. When determining the inclusion and exclusion criteria, efforts were made to ensure that the articles selected were a fair representation of all publications relevant to the research undertaken. However, because this research focused solely on peer-reviewed papers, material published on company websites, discussion forums, and other similar places could not be obtained, as previously discussed.

Failure of reviewers to extract information and data accurately and effectively from selected papers may result in data extraction errors and miscalculations. To address this issue, a combination of bibtex and JabRef, a reference management program, was used to organize and manage all the publications that we obtained for this study. The researchgate.net site is used to generate publication data in bibtex format. In addition, Microsoft Excel is also used to record and organize the extracted data items, as well as perform statistical analysis on the data.

## 5. Conclusions

Failure detection techniques on industrial machines using acoustic methods are very beneficial for the development of failure detection systems. Acoustic methods have emerged as the main means of detecting failures because of their low cost and ease of implementation.

Given the plethora of techniques, enabling technologies, and applications, it is critical to thoroughly review and analyze existing solutions to determine the degree of novelty. This SLR is an attempt to conduct a thorough review of the most recent studies on industrial engine failure detection techniques using the acoustic method. A systematic and unbiased selection process was used in 53 studies that met specific criteria for inclusion and quality of candidate studies. The findings of this study show that in a broader spectrum of acoustic failure detection methods, the use of acoustic emission remains dominant in the research community. Wear, cracks, and seeded failures continue to be the primary research topics in the context of the types of failure detected. On the other hand, the use of machine learning methods, such as SVM, k-Nearest Neighbors, artificial neural networks, and others is still the dominant choice for researchers. However, there are still challenges, such as fragility and concomitant failure, to be faced in research in this area.

According to the findings of this systematic review, several potential future research directions were also identified, including a much-needed emphasis on failure detection through the use of devices such as cell phones to process information, leading to failure recognition.

## Figures and Tables

**Table 1:** Comparison of related reviews.

References	Research Method	Year	Citations	Timeline	Focus of Study
Delvecchio et al. [24]	Traditional Review	2017	179	No	The state-of-the-art strategies and techniques based on vibroacoustic signals that can monitor and diagnose malfunctions in internal combustion engines (ICEs) under both test bench and vehicle operating conditions.
Leaman et al. [25]	Traditional Review	2021	34	No	The use of acoustic emission technology to detect failures in planetary gearboxes
Lukonge and Cao [26]	Traditional Review	2020	77	No	Utilization of acoustic emissions technology to detect offshore and onshore pipeline leaks
Raghav and Sharma [27]	Traditional Review	2020	99	No	The techniques for the condition monitoring and fault diagnosis of gearboxes based on acoustic emissions (AE)

**Table 2:** Online databases.

No	Database	URL
1	IEEE Xplore	https://ieeexplore.ieee.org/, (accessed on 24 December 2021)
2	Science Direct	https://sciencedirect.com/, (accessed on 24 December 2021)
3	Springer Link	https://link.springer.com/, (accessed on 24 December 2021)

**Table 3:** Inclusion and exclusion criteria.

Inclusion Criteria	
1	Peer-reviewed original articles
2	Articles proposing an acoustical method for mechanical failure detection
3	Articles that utilize acoustical method for failure detection
4	Recency of articles in case of multiple repeated studies
**Exclusion Criteria**	
1	Articles that are not written in English
2	Studies with unvalidated techniques and algorithms
3	Articles that utilize acoustical approach for other purposes
4	Articles that do not utilize acoustical methods
5	Articles that do not clearly mention acoustic/sound/noise approaches in the title
6	Articles providing unclear results or findings
7	Duplicated studies

**Table 4:** Data extraction.

Data Item	Description
Title	Article title
Year	Year of publication
Author(s)	The article author(s)
Publication type	Journal, proceeding, etc.
Publication medium	The medium via which the article is published
Country	Researchers’ affiliation country
Contribution	The major contribution of the article
Summary	Summary of the article from our perspective

**Table 5:** List of selected papers.

No	Authors	Year	Publication Type	Case
1	Al-Obaidi et al. [28]	2017	Journal	Valve
2	Altaf et al. [29]	2019	Journal	Rotating machine
3	Cruz et al. [30]	2020	Journal	Gas pipeline
4	Daraz et al. [31]	2018	Conference	Centrifugal Pump
5	Delgado-Prieto and Zurita Millan [32]	2017	Journal	Gear
6	Eftekharnejad and Mba [33]	2009	Journal	Gear
7	Fezari et al. [34]	2014	Conference	Rotating machine
8	Firmino et al. [35]	2021	Journal	ICE
9	Gao et al. [36]	2019	Journal	Grinder
10	Gil et al. [37]	2019	Conference	Bearing
11	Glowacz and Glowacz [38]	2017	Journal	Induction Motor
12	Glowacz et al. [39]	2021	Journal	Grinder
13	Griffin et al. [40]	2021	Journal	Metal Stamping
14	Gu et al. [41]	2011	Journal	Gearbox
15	Heydarzadeh et al. [42]	2017	Conference	Gearbox
16	Ibarra et al. [43]	2019	Journal	Bearing
17	Jian et al. [44]	2013	Journal	Bearing
18	Jo et al. [45]	2020	Journal	Turbine blade
19	Karabacak and Ozmen [46]	2021	Journal	Gear
20	Kothuru et al. [47]	2018	Journal	End Milling
21	Liu et al. [48]	2020	Journal	Gearbox
22	Liu et al. [49]	2020	Journal	Belt conveyor
23	Liu et al. [50]	2021	Journal	Turbine blade
24	Lu et al. [51]	2021	Journal	Gearbox
25	Mad Juhani and Ibrahim [52]	2016	Conference	Control valve
26	Medina et al. [53]	2019	Conference	Gear
27	Merizio et al. [54]	2021	Journal	Pipe
28	Motahari Nezad and Jafari [55]	2020	Journal	Bearing
29	Nirwan and Ramani [56]	2021	Journal	Bearing
30	Oh et al. [57]	2019	Conference	Gear Reducer
31	Omoregbee and Heyns [58]	2019	Journal	Bearing
32	Ono et al. [59]	2013	Conference	Motor
33	Orman et al. [60]	2015	Conference	Bearing
34	Pandya et al. [61]	2013	Journal	Bearing
35	Pan et al. [62]	2019	Journal	Motor
36	Park et al. [63]	2017	Journal	Insulator
37	Qiao et al. [64]	2020	Journal	Bearing
38	Qu et al. [65]	2013	Conference	Gearbox
39	Ramteke et al. [66]	2019	Journal	Diesel engine
40	Rzeszucinski et al. [67]	2015	Conference	Bearing
41	Seemuang et al. [68]	2018	Conference	Shaft
42	Shang et al. [69]	2017	Conference	Switchgear
43	Shukri et al. [70]	2011	Conference	Control valve
44	Sun et al. [71]	2020	Journal	Mill
45	Taha and Widiyati [72]	2010	Journal	Bearing
46	Tang et al. [73]	2021	Journal	Bearing
47	Toutountzakis et al. [74]	2005	Journal	Gear
48	Volkovas and Dulevicius [75]	2006	Journal	Turbine pump
49	Wu and Meng [76]	2006	Journal	Rotor
50	Yao et al. [77]	2021	Journal	Gear
51	Yun et al. [78]	2021	Journal	Robot arm
52	Zhang et al. [79]	2019	Journal	Bearing

**Table 6:** Medium of publication.

Medium of Publication	Reference
1st International Conference on Electrical Materials and Power Equipment	[69]
2nd International Conference on Engineering Innovation	[68]
3rd International Conference on Computer Research and Development	[70]
4th International Conference on Intelligent and Automation Systems	[52]
10th IEEE International Symposium on Diagnostics for Electrical Machines, Power Electronics and Drives	[67]
10th International Conference on Information and Communication Technology Convergence	[57]
16th International Power Electronics and Motion Control Conference and Exposition	[34]
24th International Conference on Automation & Computing	[31]
42nd IEEE International Conference on Acoustics, Speech and Signal Processing	[42]
2013 IEEE International Conference on Prognostics and Health Management	[65]
2nd International Conference on Condition Assessment Techniques in Electrical Systems	[60]
2019 Signal Processing Algorithms, Architectures, Arrangements, and Applications	[37]
2019 Prognostics and System Health Management Conference	[53]
Acoustics, Speech, and Signal Processing	[59]
Acoustic Australia	[29]
Advance Powder Technology	[49]
Alexandria Engineering Journal	[28]
Applied Acoustic	[33,38,39,73]
Chinese Journal of Mechanical Engineering	[36,51]
Clean Technologies and Environmental Policy	[30]
Expert Systems with Application	[61]
IEEE Access	[64]
IEEE Sensors Journal	[48]
IEEE Transactions on Industrial Electronics	[32,63]
IEEE Transactions on Industry Applications	[50]
IEEE Transactions on Instrumentation and Measurement	[77]
International Journal of Advanced Manufacturing Technology	[76]
International Journal of Precision Engineering and Manufacturing	[44]
Journal of Intelligent Manufacturing	[78]
Journal of Mechanical Science and Technology	[41,45,62,79]
Journal of the Brazilian Society of Mechanical Sciences and Engineering	[35]
Journal of The Institution of Engineers (India): Series C	[54]
Journal of Vibration Engineering & Technologies	[58,66]
Material Today: Proceedings	[56]
Measurement	[46,55]
NDT & E International	[74]
Russian Journal of Nondestructive Testing	[75]
The International Journal of Advanced Manufacturing Technology	[40,43,47,71,72]

**Table 7:** Types of failure detected by acoustic method.

Failure	Location	Accuracy	Reference
Burn	Grinder	≤100%	[36]
Breakage	Milling Machine	91.18%	[71]
Corrosion	Valve	98%	[28]
Crack	Bearing	80–100%	[72]
		-	[44]
	Gear	97%	[57]
		-	[48]
	Propeller	-	[75]
	Shaft	-	[68]
Fracture	Gear	≥90%	[77]
		72%	[32]
Leakage	Pipeline	99.6%	[30]
	Control Valve	-	[52]
Misfire	Combustion Engine	98.7–99.3%	[35]
Pitting	Gear	97.0–99.9%	[53]
Rubbing	Motor	80%	[62]
Wear	Bearing	56.3–100%	[55]
		-	[43]
	Gear	48.4–99.9%	[46]
		-	[51,65]
	Metal Stamping	96%	[40]
	Other	97%	[47]
Seeded	Bearing	96.67%	[61]
		-	[60]
	Gear	-	[33,74]
Spall	Bearing	-	[67]
Another Failure	Bearing	89.33–100%	[39]
		87.2–99.48%	[64]
		-	[29,37,50,56,58,73,76,79]
	Pipe	100%	[54]
	Turbine Blade	-	[45]
	Insulator	96.7–100%	[63]
	Belt Conveyor	94.53%	[49]
	Diesel Engine	-	[66]
	Centrifugal Pump	-	[31]
	Control Valve	-	[70]
	Motor	82–100%	[59]
		-	[38]
	Robot Arm	85%	[78]
	Rotataing Machine	91.5–94.5%	[34]
	Gear	97%	[42]
		-	[41]
	Switchgear	-	[69]

**Table 8:** Approach methods used to detect failures based on acoustic signals.

Detection Method	Analysis	Reference
Acoustic Emission	Adaptive Neuro-Fuzzy Inference System	[55]
	Akaike Information Criterion	[73]
	Cepstrum	[43,44]
	Chromatic monitoring	[32]
	Envelope	[41]
	Frequency	[52]
	Machine Learning	[28,34,40,42,53,58,61,71,72]
	Root Mean Square	[33,56,68,74]
	Sparse Augmented Lagrangian	[50]
	Statistic	[62,66,70,75]
	Time Synchronous Average	[65]
	Variational Mode Decomposition	[48]
	Wavelet	[36]
Microphone	Envelope	[31]
	Modulation Signal Bispectrum	[51]
	Machine Learning	[29,30,35,37,38,46,47,49,54,57,63,64,77,78]
	Reverse Spectrum	[69]
	Shortened Method of Frequency Selection Nearest Frequency Components	[39]
	Special Kurtosis	[60,67]
	Statistic	[59]
	Stochastic Resonance	[79]
	Time-frequency	[76]
Ultrasonic	Quantitative	[45]

**Table 9:** Algorithm or analysis method used to define failure.

Intelligent	Clasical
Adaptive Neuro-Fuzzy Inference System	High-Order Statistics
Support Vector Machine (SVM)	Akaike Information Criterion
Decision Tree	Mel-Frequency Cepstral Coefficients
Classification and Regression Tree	Sparse Augmented Lagrangian
Genetic Algorithm	Variational Mode Decomposition
k-Nearest Neighbors (KNN)	Cepstrum Pre-Whitening
Kernel Liner Discriminant Analysis	Special Kurtosis
Negative Selection Algorithm	Envelope Analysis
Recursive Denoising Learning	Time-Frequency Analysis
Random Forest (RF)	Modulation Signal Bispectrum
Neural Network	
Sparse Discriminant Analysis	

**Table 10:** Summary of the technical implementation of artificial intelligence aspect in mechanical failure detection.

Author	Failure Location	Algorithm	Dataset	Environment
Al-Obaidi et al. [28]	Valve	SVM	142,035 samples of AE signal statistical parameters	Laboratory
Altaf et al. [29]	Rotating Machine	SVM, kernel liner discriminant analysis, KNN, sparse discriminant analysis	Audible sound frequency ranges from 20 Hz to 20 KHz	Laboratory
Cruz et al. [30]	Gas Pipeline	Logistic regression, KNN, SVM with linear kernel, SVM with radial basis kernel, random forest, adaptive boosting, extreme gradient boosting	1680 samples (120 samples for each of the 14 experiments) and for regression of 840 samples (120 samples for each of the leakage experiments) in 7 orifices	Laboratory
Fezari et al. [34]	Rotating Machine	K-Nearest Neighbors	10 recordings of 5 s duration with frequency sampling $F_{s}=$ 10,000 Hz	Laboratory
Firmino et al. [35]	Internal Combustion Engine	Artificial neural network	Frequencies, amplitudes, and energy data gathered using acoustic acquisition system	Laboratory
Griffin et al. [40]	Metal Stamping	Classification and regression tree	A reduced short-time Fourier transform of top 10 absolute maximum component AE feature sets that correlates to wear measurement data	Laboratory
Heydarzadeh et al. [42]	Gearbox	SVM	Recording of gearbox acoustic emissions using an open field microphone at the rate of 5 KHz for 5 load conditions and four classes corresponding to fault-free, pinion, wheel, and simultaneous faults	Laboratory
Karabacak and Ozmeri [46]	Gear	Artificial neural network	Artificially produced acoustic signal samples on machines that have failures caused by wear, pitting, and breakage	Laboratory
Kothuru et al. [47]	End Milling	SVM	Audio signal related to wear level	Laboratory
Liu et al. [49]	Belt Conveyor	Decision tree	42 sets of acoustic data acquired from experiments with a belt velocity of 1 m/s, which is equivalent to 2.9 rpm for the idler rolls	Laboratory
Medina et al. [53]	Gear	Long short-term memory	Acoustic emission signal datasets	Laboratory
Merizio et al. [54]	Pipe	Negative selection algorithm	Collection of sound pressure data in positions inside the tube using ISO10534-1(1996) standard	Laboratory
Motahari Nezad and Jafari [55]	Bearing	Adaptive neuro-fuzzy inference system	Acoustic emission signals	Laboratory
Oh et al. [57]	Gear Reducer	SVM	A balanced data set of 300 acoustic signals to accommodate four cases of 60 signals and 60 signals each in normal operation	Laboratory
Omoregbee and Heyns [58]	Bearing	SVM, and genetic algorithm	A GA-based feature extractor from a raw acoustic emission dataset	Laboratory
Pandya et al. [61]	Bearing	Asymmetric proximity function KNN	180 data samples of the five bearing conditions	Laboratory
Park et al. [63]	Insulator	Neural network	Samples of noise measurement results on insulators	Laboratory
Qiao et al. [64]	Bearing	CNN, long short-term memory	Data of 10 different fault levels, including inner race, outer race, ball, and normal. Each fault type collects 800 samples, and 1200 signal points make a group of samples	Noisy
Sun et al. [71]	Mill	SVM	Acoustic signal samples from the engine during operation for normal and abnormal conditions	Laboratory
Taha and Widiyati [72]	Bearing	Artificial neural network	Acoustic signal samples from five bearing defect conditions	Laboratory
Yao et al. [77]	Gear	Recursive denoising learning	The collection of clean acoustic signal and noise-disturbed acoustic signal	Laboratory
Yun et al. [78]	Robot Arm	Neural network	A collection of acoustic signal samples measured at each joint	Laboratory

**Table 11:** Challenges in acoustic-based detection.

Challenges	Explanation
Environmental noise	The type of noise is very influential on the measurement results. Noise dominated by impulse signals will certainly make failure analysis difficult because the spectrum of the signal will be present and affect all observed frequencies.
Fragility	Failure is very likely to occur in components that are already fragile. Failures such as defects or leaks can be detected, but because there is a tendency to change the size of the defect level in a short time, the measurement results will vary.
Multivariate failures	Failures that occur in a machine can come from several points and occur at the same time. In addition, the type of failure that occurs can also be a mixture of defects, cracks, leaks, wear, and others. Each failure will affect the measurement signal received and will affect the failure analysis method used.
Concurrent failure	Failure may occur on more than one machine running at the same time. The sensor will be very easily affected by interference signals from equipment around the measuring object that also fails, especially for microphone-based measurements.

## References

[1] Fan L., Zhang F., Fan H., Zhang C. (2019). Brief review of image denoising techniques. Vis. Comput. Ind. Biomed. Art.

[2] Ali M.H., Jaber M.M., Abd S.K., Rehman A., Awan M.J., Vitkutė-Adžgauskienė D., Damaševičius R., Bahaj S.A. (2022). Harris Hawks Sparse Auto-Encoder Networks for Automatic Speech Recognition System. Appl. Sci..

[3] Butkeviciute E., Bikulciene L., Sidekerskiene T., Blazauskas T., Maskeliunas R., Damasevicius R., Wei W. (2019). Removal of Movement Artefact for Mobile EEG Analysis in Sports Exercises. IEEE Access.

[4] Damasevicius R., Napoli C., Sidekerskiene T., Wozniak M. (2017). IMF mode demixing in EMD for jitter analysis. J. Comput. Sci..

[5] Picaut J., Can A., Fortin N., Ardouin J., Lagrange M. (2020). Low-cost sensors for urban noise monitoring networks—A literature review. Sensors.

[6] Kantová R. (2021). Evaluation of Construction Site Noise to Allow the Optimisation of Construction Processes and Construction Machinery Selection. Appl. Sci..

[7] Ahmed S.S., Gadelmoula A.M. (2022). Industrial noise monitoring using noise mapping technique: A case study on a concrete block-making factory. Int. J. Environ. Sci. Technol..

[8] Lv Y., Liu Y., Jing W., Woźniak M., Damaševičius R., Scherer R., Wei W. (2020). Quality control of the continuous hot pressing process of medium density fiberboard using fuzzy failure mode and effects analysis. Appl. Sci..

[9] Araújo Alves J., Neto Paiva F., Torres Silva L., Remoaldo P. (2020). Low-Frequency Noise and Its Main Effects on Human Health—A Review of the Literature between 2016 and 2019. Appl. Sci..

[10] Paar R., Marendić A., Jakopec I., Grgac I. (2021). Vibration monitoring of civil engineering structures using contactless vision-based low-cost iats prototype. Sensors.

[11] Weintroub S. (1960). Noise in Factories and its Control. Nature.

[12] Bi X. (2020). Infrared Sensors and Ultrasonic Sensors.

[13] Silva C.W. (2007). Sensors and Actuators: Control System Instrumentation.

[14] Leukel J., González J., Riekert M. (2021). Adoption of machine learning technology for failure prediction in industrial maintenance: A systematic review. J. Manuf. Syst..

[15] Hua X., Ono Y., Peng L., Cheng Y., Wang H. (2021). Target Detection Within Nonhomogeneous Clutter Via Total Bregman Divergence-Based Matrix Information Geometry Detectors. IEEE Trans. Signal Process..

[16] Maio A. (2019). Invariance Theory for Adaptive Radar Detection in Heterogeneous Environment. IEEE Signal Process. Lett..

[17] Levikari S., Kärkkäinen T., Andersson C., Tamminen J., Nykyri M., Silventoinen P. (2020). Nondestructive Acoustic Testing of Ceramic Capacitors Using One-Class Support Vector Machine With Automated Hyperparameter Selection. IEEE Access.

[18] Simonovic M., Kovandzic M., Ciric I., Nikolic V. (2021). Acoustic Recognition Of Noise-like Environmental Sounds by Using Artificial Neural Network. Expert Syst. Appl..

[19] Molina Vicuna C., Howeler C. (2017). A method for reduction of Acoustic Emission (AE) data with application in machine failure detection and diagnosis. Mech. Syst. Signal Process..

[20] Holford K., Eaton M., Hensman J., Pullin R., Evans S., Dervilis N., Worden K. (2017). A new methodology for automating acoustic emission detection of metallic fatigue fractures in highly demanding aerospace environments: An overview. Prog. Aerosp. Sci..

[21] Cooper C., Wang P., Zhang J., Gao R., Roney T., Ragai I., Shaffer D. (2020). Convolutional neural network-based tool condition monitoring in vertical milling operations using acoustic signals. Procedia Manuf..

[22] Tagawa Y., Maskeliunas R., Damasevicius R. (2021). Acoustic Anomaly Detection of Mechanical Failures in Noisy Real-Life Factory Environments. Electronics.

[23] Reubens J., Verhelst P., van der Knaap I., Deneudt K., Moens T., Hernandez F. (2019). Environmental factors influence the detection probability in acoustic telemetry in a marine environment: Results from a new setup. Hydrobiologia.

[24] Bonfiglio P., Pompoli F., Delvecchio S. (2018). Vibro-acoustic condition monitoring of Internal Combustion Engines: A critical review of existing techniques. Mech. Syst. Signal Process..

[25] Leaman F., Clausen E., Molina Vicuna C. (2021). A Review of Gear Fault Diagnosis of Planetary Gearboxes Using Acoustic Emissions. Acoust. Aust. / Aust. Acoust. Soc..

[26] Lukonge A., Cao X. (2020). Leak Detection System for Long-Distance Onshore and Offshore Gas Pipeline Using Acoustic Emission Technology. A Review. Trans. Indian Inst. Met..

[27] Raghav M., Sharma R. (2020). A Review on Fault Diagnosis and Condition Monitoring of Gearboxes by Using AE Technique. Arch. Comput. Methods Eng..

[28] Al-Obaidi S., Hui K.H., Hee L., Leong M. (2017). Automated valve fault detection based on acoustic emission parameters and support vector machine. Alex. Eng. J..

[29] Altaf M., Khan M., Ahmad A., Badshah S., Shah J., Anjum M.A. (2019). Automatic and Efficient Fault Detection in Rotating Machinery using Sound Signals. Acoust. Aust..

[30] Cruz R., Silva F., Fileti A. (2020). Machine learning and acoustic method applied to leak detection and location in low-pressure gas pipelines. Clean Technol. Environ. Policy.

[31] Daraz A., Alabied S., Smith A., Gu F., Ball A. Detection and Diagnosis of Centrifugal Pump Bearing Faults Based on the Envelope Analysis of Airborne Sound Signals. Proceedings of the 2018 24th International Conference on Automation and Computing (ICAC).

[32] Delgado-Prieto M., Zurita Millan D. (2017). Chromatic Monitoring of Gear Mechanical Degradation Based on Acoustic Emission. IEEE Trans. Ind. Electron..

[33] Eftekharnejad B., Mba D. (2009). Seeded fault detection on helical gears with acoustic emission. Appl. Acoust..

[34] Fezari M., Taif F., Lafifi M.M. Noise emission analysis a way for early detection and classification faults in rotating machines. Proceedings of the 2014 16th International Power Electronics and Motion Control Conference and Exposition.

[35] Firmino J., Neto J., Oliveira A., Silva J., Mishina K., Rodrigues M. (2021). Misfire detection of an internal combustion engine based on vibration and acoustic analysis. J. Braz. Soc. Mech. Sci. Eng..

[36] Gao Z., Lin J., Wang X., Yuhe L. (2019). Grinding Burn Detection Based on Cross Wavelet and Wavelet Coherence Analysis by Acoustic Emission Signal. Chin. J. Mech. Eng..

[37] Gil D., Grochowina M., Leniowska L. Detecting of the rolling bearing state based on acoustic signal and the k-NN classifier. Proceedings of the 2019 Signal Processing: Algorithms, Architectures, Arrangements, and Applications (SPA).

[38] Glowacz A., Glowacz Z. (2017). Diagnosis of stator faults of the single-phase induction motor using acoustic signals. Appl. Acoust..

[39] Glowacz A., Tadeusiewicz R., Legutko S., Caesarendra W., Irfan M., Liu H., Brumercik F., Gutten M., Sułowicz M., Antonino-Daviu J. (2021). Fault diagnosis of angle grinders and electric impact drills using acoustic signals. Appl. Acoust..

[40] Griffin J., Shanbhag V., Pereira M., Rolfe B. (2021). Application of machine learning for acoustic emissions waveform to classify galling wear on sheet metal stamping tools. Int. J. Adv. Manuf. Technol..

[41] Gu D., Kim J., An Y., Choi B.K. (2011). Detection of faults in gearboxes using acoustic emission signal. J. Mech. Sci. Technol..

[42] Heydarzadeh M., Nourani M., Hansen J., Kia S. Non-invasive Gearbox Fault Diagnosis Using Scattering Transform of Acoustic Emission. Proceedings of the 2017 IEEE International Conference on Acoustics, Speech and Signal Processing (ICASSP).

[43] Ibarra D., Tamayo O., Vallejo-Guevara A. (2019). Bearing fault diagnosis in rotating machinery based on cepstrum pre-whitening of vibration and acoustic emission. Int. J. Adv. Manuf. Technol..

[44] Jian H., Lee H.R., Ahn J.H. (2013). Detection of Bearing/Rail Defects for Linear Motion Stage Using Acoustic Emission. Int. J. Precis. Eng. Manuf..

[45] Jo H., Kim Y., Jo D. (2020). Acoustically monitoring defects on rotating turbine blades. J. Mech. Sci. Technol..

[46] Karabacak Y., Ozmen N. (2021). Common Spatial Pattern-based Feature Extraction and Worm Gear Fault Detection through Vibration and Acoustic Measurements. Measurement.

[47] Kothuru A., Nooka S., Liu R. (2018). Application of audible sound signals for tool wear monitoring using machine learning techniques in end milling. Int. J. Adv. Manuf. Technol..

[48] Liu L., Chen L., Wang Z., Liu D. (2020). Early Fault Detection of Planetary Gearbox Based on Acoustic Emission and Improved Variational Mode Decomposition. IEEE Sens. J..

[49] Liu X., Pei D., Lodewijks G., Zhao Z., Mei J. (2020). Acoustic signal based fault detection on belt conveyor idlers using machine learning. Adv. Powder Technol..

[50] Liu Z., Yang B., Wang X., Zhang L. (2021). Acoustic Emission Analysis for Wind Turbine Blade Bearing Fault Detection Under Time-Varying Low-Speed and Heavy Blade Load Conditions. IEEE Trans. Ind. Appl..

[51] Lu K., Gu J., Fan H., Sun X., Li B., Gu F. (2021). Acoustics Based Monitoring and Diagnostics for the Progressive Deterioration of Helical Gearboxes. Chin. J. Mech. Eng..

[52] Mad Juhani J., Ibrahim R. Acoustic emission technique for early leakage detection of in-service control valve. Proceedings of the 2016 6th International Conference on Intelligent and Advanced Systems (ICIAS).

[53] Medina R., Cerrada M., Cabrera D., Sánchez R., Li C., de Oliveira J. Deep Learning-Based Gear Pitting Severity Assessment Using Acoustic Emission, Vibration and Currents Signals. Proceedings of the 2019 Prognostics and System Health Management Conference (PHM-Paris).

[54] Merizio I., Chavarette F., Moro T., Outa R., Mishra V. (2021). Machine Learning Applied in the Detection of Faults in Pipes by Acoustic Means. J. Inst. Eng. India Ser. C.

[55] Motahari Nezhad M., Jafari S. (2020). ANFIS System for Prognosis of Dynamometer High-Speed Ball Bearing Based on Frequency Domain Acoustic Emission Signals. Measurement.

[56] Nirwan N., Ramani H.B. (2021). Condition monitoring and fault detection in roller bearing used in rolling mill by acoustic emission and vibration analysis. Mater. Today Proc..

[57] Oh S.W., Lee C., You W. Gear Reducer Fault Diagnosis Using Learning Model for Spectral Density of Acoustic Signal. Proceedings of the 2019 International Conference on Information and Communication Technology Convergence (ICTC).

[58] Omoregbee H., Heyns S. (2019). Fault Classification of Low-Speed Bearings Based on Support Vector Machine for Regression and Genetic Algorithms Using Acoustic Emission. J. Vib. Eng. Technol..

[59] Ono Y., Onishi Y., Koshinaka T., Takata S., Hoshuyama O. Anomaly detection of motors with feature emphasis using only normal sounds. Proceedings of the 2013 IEEE International Conference on Acoustics, Speech and Signal Processing.

[60] Orman M., Rzeszucinski P., Tkaczyk A., Krishnamoorthi K., Pinto C., Sułowicz M. Bearing fault detection with the use of acoustic signals recorded by a hand-held mobile phone. Proceedings of the 2015 International Conference on Condition Assessment Techniques in Electrical Systems (CATCON).

[61] Pandya D., Upadhyay S., HARSHA S. (2013). Fault diagnosis of rolling element bearing with intrinsic mode function of acoustic emission data using APF-KNN. Expert Syst. Appl..

[62] Pan Q., Zhou R., Su J., He T., Zhang Z. (2019). Automatic localization of the rotor-stator rubbing fault based on acoustic emission method and higher-order statistics. J. Mech. Sci. Technol..

[63] Park K.C., Motai Y., Yoon J. (2017). Acoustic Fault Detection Technique for High Power Insulators. IEEE Trans. Ind. Electron..

[64] Qiao M., Yan S., Tang X., Xu C. (2020). Deep Convolutional and LSTM Recurrent Neural Networks for Rolling Bearing Fault Diagnosis Under Strong Noises and Variable Loads. IEEE Access.

[65] Qu A., Zhu J., He D., Qiu B., Bechhoefer E. Development of a new acoustic emission based fault diagnosis tool for gearbox. Proceedings of the 2013 IEEE International Conference on Prognostics and Health Management, PHM 2013.

[66] Ramteke S., Chelladurai H., Muniyappa A. (2019). Diagnosis of Liner Scuffing Fault of a Diesel Engine via Vibration and Acoustic Emission Analysis. J. Vib. Eng. Technol..

[67] Rzeszucinski P., Orman M., Pinto C., Tkaczyk A., Sulowicz M. A signal processing approach to bearing fault detection with the use of a mobile phone. Proceedings of the 2015 IEEE 10th International Symposium on Diagnostics for Electrical Machines, Power Electronics and Drives (SDEMPED).

[68] Seemuang N., Lim J., Kaewkongka T. Shaft Crack Monitoring by Using Acoustic Emission Technique. Proceedings of the 2018 2nd International Conference on Engineering Innovation (ICEI).

[69] Shang Y., Liu Q., Niu B., Zhang M., Qi W., Wu J. Mechanical fault diagnosis system based on acoustic feature analysis in gas insulated switchgear. Proceedings of the 2017 1st International Conference on Electrical Materials and Power Equipment (ICEMPE).

[70] Shukri I., Mun G., Ibrahim R. A study on control valve fault incipient detection monitoring system using Acoustic Emission technique. Proceedings of the 2011 3rd International Conference on Computer Research and Development.

[71] Sun S., Hu X., Zhang W. (2020). Detection of tool breakage during milling process through acoustic emission. Int. J. Adv. Manuf. Technol..

[72] Taha Z., Widiyati K. (2010). Artificial neural network for bearing defect detection based on acoustic emission. Int. J. Adv. Manuf. Technol..

[73] Tang L., Liu X., Wu X., Wang Z., Hou K. (2021). Defect localization on rolling element bearing stationary outer race with acoustic emission technology. Appl. Acoust..

[74] Toutountzakis T., Tan C., Mba D. (2005). Application of Acoustic Emission to Seeded Gear Fault Detection. NDT E Int..

[75] Volkovas V., Dulevicius J. (2006). Acoustic emission used for detection of crack generation in propellers of turbine-pump units. Russ. J. Nondestruct. Test..

[76] Wu F., Meng G. (2006). Feature extraction based on the 3D spectrum analysis of acoustic signals to identify rotor malfunction. Int. J. Adv. Manuf. Technol..

[77] Yao Y., Gui G., Yang S., Zhang S. (2021). A Recursive Denoising Learning for Gear Fault Diagnosis Based on Acoustic Signal in Real-Industrial Noise Condition. IEEE Trans. Instrum. Meas..

[78] Yun H., Kim H., Jeong Y., Jun M. (2021). Autoencoder-based anomaly detection of industrial robot arm using stethoscope based internal sound sensor. J. Intell. Manuf..

[79] Zhang J., Yang J., Litak G., Hu E. (2019). Feature extraction under bounded noise background and its application in low speed bearing fault diagnosis. J. Mech. Sci. Technol..

[80] Yang C., Mariton M. Machine failure detection in manufacturing systems. Proceedings of the 1994 33rd IEEE Conference on Decision and Control.

